# Residual Stress Relief in 2219 Aluminium Alloy Ring Using Roll-Bending

**DOI:** 10.3390/ma13010105

**Published:** 2019-12-24

**Authors:** Hai Gong, Xiaoliang Sun, Yaoqiong Liu, Yunxin Wu, Yanan Wang, Yanjie Sun

**Affiliations:** 1 Light Alloy Research Institute, Central South University, Changsha 410083, China; xiaoliang_sun163@163.com (X.S.); wuyunxin@csu.edu.cn (Y.W.); sunyanjie@csu.edu.cn (Y.S.); 2 State Key Laboratory of High Performance Complex Manufacturing, Central South University, Changsha 410083, China; yaoqiongliu_11@163.com (Y.L.); wyn-15@csu.edu.cn (Y.W.); 3 College of Mechanical and Electrical Engineering, Central South University, Changsha 410083, China; 4 Institute of Standard Materials Technology, Avic the First Aircraft Institute, Xi’an 710000, China

**Keywords:** roll-bending, 2219 Al alloy ring, residual stress, three-roller bending beam

## Abstract

Relieving the residual stress in components is essential to improve their service performance. In this study, a roll-bending process was proposed to reduce the quenching residual stress in a large-size 2219 Al alloy ring. The roll-bending effect on quenching residual stress was evaluated via the finite element method (FEM) combined with experiment. The effect of radial feed quantity, friction coefficient, and roller rotational speed during the roll-bending process on quenching residual stress was analyzed. A set of optimized roll-bending parameters with radial feed quantity, friction coefficient, and roller rotational speed was obtained. The results reveal that the best reduction rates of circumferential and axial residual stress reached 61.72% and 86.24%, respectively. Furthermore, the difference of the residual stress reduction effect between the roll-bended ring and the three-roller bended beam was analyzed.

## 1. Introduction

In the aerospace field, the requirement of material properties is very high due to the extremely complex service conditions. Therefore, improving the overall properties of the alloy is of great significance. The 2219 Al alloy is widely used in fuel tanks of launch vehicles. In addition to its high strength, 2219 Al possesses good fracture toughness and excellent stress corrosion resistance [1]. The 2219 Al alloy is usually treated by solution heat treatment and quenching to improve its overall performance before the aforementioned components are manufactured. The process is applied to large-size 2219 Al alloy rings with a diameter up to 5000 mm [2,3]. While improving the mechanical performance of the material, as described by Prime [4], an inevitable detrimental residual stress is induced in the material. Usually, the residual stress is introduced into the ring during quenching process as described by Singh and Agrawal [5]. The components become vulnerable during subsequent machining, as described by Koç [6], which adversely affects the manufacturing process. Therefore, reducing the residual stress is essential to enhance the performance of the ring. In the study of Cui [7], distortion, crack nucleation, and propagation resulting from the subsequent machining processes, which may accelerate the ring’s failure, will be avoided by reducing the large residual stress. 

Generally, the residual stress reduction method can be divided into two types, namely, a thermal action method and a mechanical load method (cold-working method). Younger and Eckelmeyer [8] analyzed the effectiveness of heat and aging treatment on the residual stress of a heat-treated Al alloy satellite box. The results were compared with cold working techniques, it was reported that the aging treatment (thermal method) only reduces the residual stress magnitude by 40%, while it led to a decrease in key material properties such as tensile strength and stiffness. Other methods, such as shot peening for AlSi10Mg, vibratory stress relief for 7075 Al alloy, uphill quenching treatment for 6061 Al alloys, multi-stage quenching for 7050 Al alloy, and quenchant changing technique for 2000, 6000, and 7000 series Al alloys, have been implemented to relax residual stress by Maamoun et al. [9], Gong et al. [10], Jones [11], Zhang et al. [12], and Robinson et al. [13], respectively. Although these methods are effective in relieving residual stress in small components. Hence, applying these methods in large components would be a bit complex and costly, considering the cost of materials, availability of required equipment and capabilities. Pan et al. [14] studied the residual stress of 7050 Al alloy forging after quenching, the result indicated that the cold rolling process almost eliminated the tensile residual stress in the component. Tanner and Robinson [15] investigated the effectiveness of cold compression and cold stretching on the residual stress distribution of heat-treated components. The result indicated that these two methods could significantly mitigate the quenching-induced residual stress magnitude by about 90%. Cozzolino and Luis [16] studied the residual stress of the steel plate after welding, and local rolling treatment was used to reduce the welding residual stress. The research indicated that local rolling could effectively reduce the residual stress introduced by welding. Moreover, the influence of rolling parameters such as friction coefficient and roller type on the effect of residual stress reduction was discussed. Compared to other methods, cold rolling is cost-effective and versatile. In addition, the surface of components is high quality after cold rolling. Cold rolling has a short process cycle and more efficient, and it can be applied in relieving residual stress in heat-treated extra-long components. 

While the traditional residual stress reduction methods are not suitable for ring components, limited by the structural characteristics of rings and existing equipment conditions. Roll-bending is generally considered as a forming technique for continuous cold rolling as described in Hua [17]. Pan et al. [18] researched the thermal residual stresses relaxation in AA7xxx Al alloy through cold rolling. The result revealed that cold rolling transforms near-surface residual stresses from large compression to large tension along the rolling direction. And Abvabi et al. [19] investigated the influence of residual stress on a roll bending forming process of DP780 steel plates. The result indicated that a thickness reduction rolling process led to the introduction of tensile residual stress at the material surface, and compressive residual stress in the mid-plane of the plate.

In this study, a roll-bending process was proposed to reduce the residual stress of large-size 2219 Al alloy rings. Then, the effect of roll-bending on residual stress relief was evaluated. Numerical simulation and experiment were carried out to analyze the performance of the roll-bending process on 2219 Al alloy ring. The influence of the radial feed quantity, friction coefficient, and rotational speed were analyzed. Finally, a set of optimized roll-bending parameters for reducing residual stress of the ring was proposed. The roll-bending method with optimized parameters proposed in this study has been successfully applied to residual stress relief in large-size 2219 Al alloy ring. After residual stress relief, the ring will be used in the subsequent manufacturing process for launch vehicles fuel tanks.

## 2. Material and Methods

The chemical compositions (wt.%) of 2219 Al alloy used in this study are shown in Table 1.

**Figure 1:**
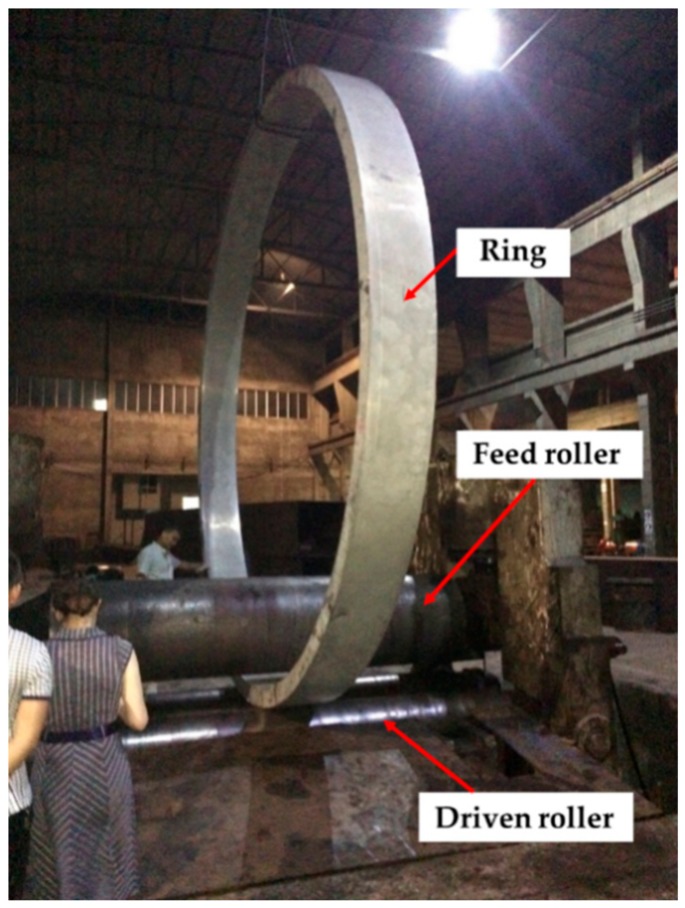
Roll-bending experiment.

Large-size 2219 Al alloy ring with outer diameter 5000 mm, inner diameter 4700 mm, and height 410mm was studied in this work. The ring was solution heat treated at 813 K for about 8 h, then quenched in water, and finally roll-bended. The roll-bending device consist of three rollers, with the feed roller located at the top and two driven rollers at the bottom as shown in Figure 1. The diameters of the feed roller and driven roller are 770 mm and 460 mm, respectively. The maximum processing thickness of the roll-bending machine is about 200 mm. The center distance between the two driven rollers is 800 mm. Maximum load of the feed roller in roll-bending process is about 1000 tons.

**Figure 2:**
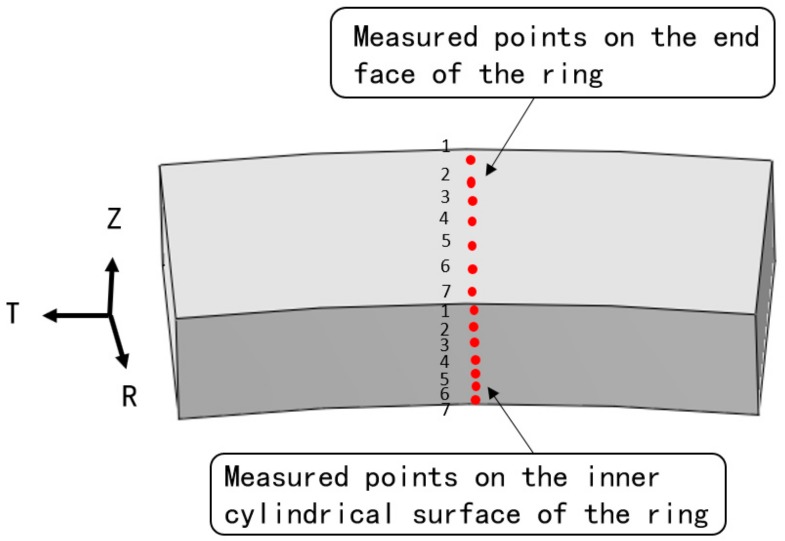
Residual stress measurement area.

Surface residual stress of the large ring was measured using X-Ray Diffractometer (XRD, STRESSTECH OY, Jyväskylä, Finland), and the measurement result was compared with FEM result. The measurement area was shown in Figure 2. The measurement was carried out in steps of 10 mm interval in the radial and axial direction. The diffractometer parameters used in the measurement was shown in Table 2.

## 3. Numerical Modelling of the Quenching and Roll-Bending Process of the Ring

The residual stress analysis based on thermo-elastic-plastic (TEP) FEM was usually applied. In this section, the quenching and roll-bending process numerical modelling of the ring was established.

### 3.1. Numerical Modelling of 2219 Ring Quenching

The finite element model of the ring quenching process was established using ABAQUS software (the version number: ABAQUS 6.14). The eight-node linear hexahedral elements (HEX C3DR8T) were assigned to the whole model, and a coupled temperature-displacement analysis was performed. The thermo-mechanical properties of the material used are shown in Table 3. In the finite element model, the Cartesian coordinate system was converted into a cylindrical coordinate system, and the R, T, and Z coordinates in the cylindrical coordinate system correspond to the radial, circumferential, and axial directions of the ring respectively.

The following assumptions were adopted in the quenching model:

(1) The material of the ring is continuous and isotropic.
(2) The initial temperature field distribution of the ring is uniform, and the initial residual stress inside the ring is negligible.
(3) The temperature of the quenching medium remains uniform.
(4) The phase change of the ring during the quenching process is not considered.

### 3.2. Numerical Modelling of 2219 Ring Roll-Bending

After quenching, the roll-bending process of the ring was simulated by taking the calculated quenching residual stress as the predefined initial stress, then, the effects of radial feed quantity (*L*), friction coefficient (*f*) and roller rotational speed (*v*) during the roll-bending on quenching residual stress were analyzed.

The following assumptions were adopted in the roll-bending model:

(1) The material of the ring is continuous and isotropic.
(2) The friction coefficient between the ring and the feed roller and between the ring and the driven roller is constant during the same roll-bending process.
(3) The effect of vibration on the overall analysis results is neglected.

**Figure 3:**
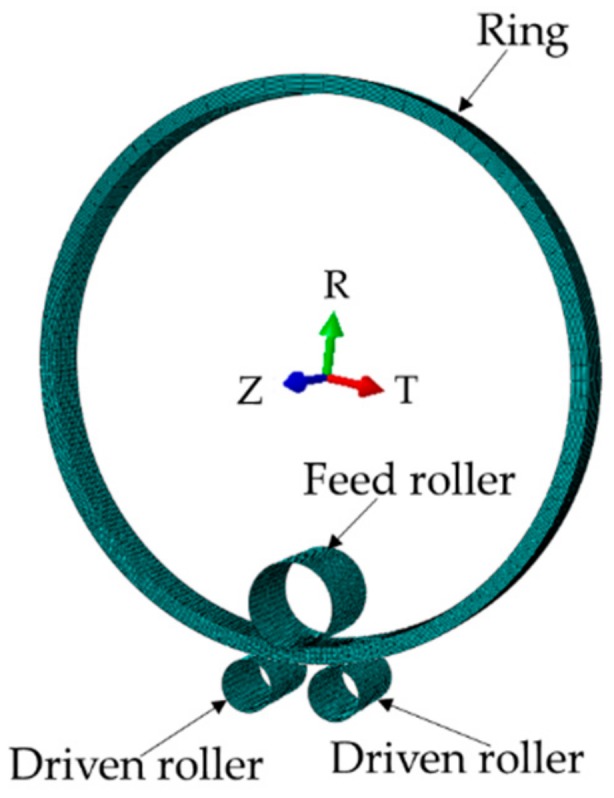
Finite model of ring roll-bending.

**Figure 4:**
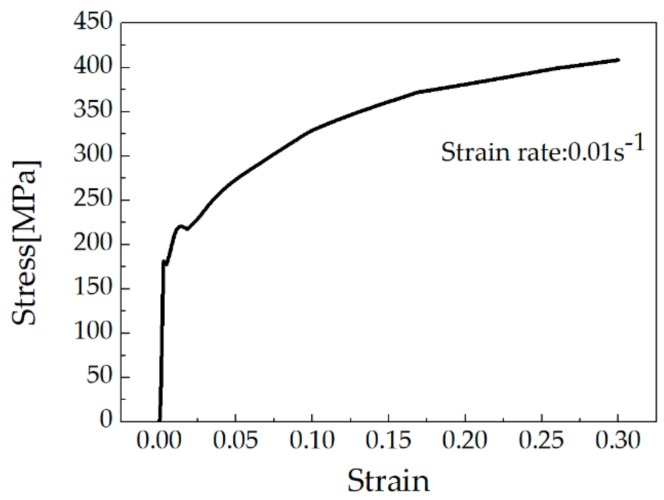
Mechanical properties of Al 2219 during the plastic deformation.

The roll-bending model is shown in Figure 3. A symmetrical three-axle roller-bending machine was adopted. The feed roller is located at the top with diameter 770 mm and height 800 mm. The two driven rollers are located at the bottom with diameter 460 mm and height 800 mm. The material mechanical parameters used in the model are shown in Figure 4.

The roll-bending process was divided into three stages. In the first stage, the feed roller was moved downward by 12 mm in the radial direction, while both driven rollers remain stationary. In the second stage, the two driven rollers rotate around the axial direction, and the influence of the frictional force causes the ring to rotate, thereby driving the feed roller to rotate. The speed of the driven roller was set as 0.3 rad/s, and the speed in other directions was not limited. The boundary condition applied to the driven roller is that the axial rotational displacement is not limited, and the displacements of the other five directions are set to zero. In the third stage, the feed roller was moved 100 mm radially away from the center of the ring, and displacements in the other five directions are set to zero. While the driven roller remains stationary, limiting its free displacement.

## 4. Numerical Analysis of the Quenching and Roll-Bending Process of the Ring

**Figure 5:**
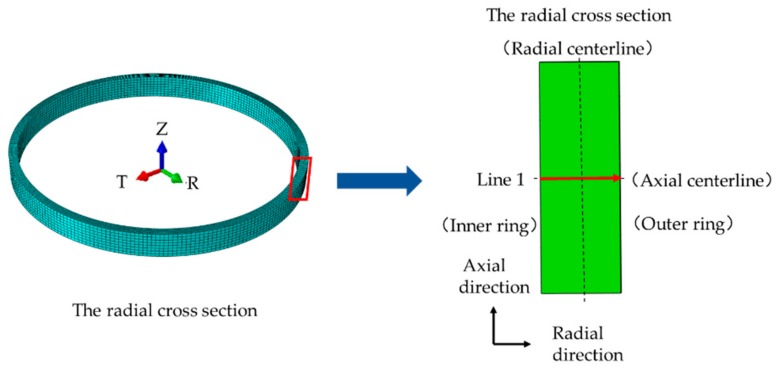
Section for ring residual stress analysis.

According to the structural characteristic of the large ring, a section was selected for residual stress analysis. The cross section is shown in Figure 5.

It was found in this study that the radial residual stress of the ring is relatively small, while the circumferential and axial stress are much bigger. Therefore, the circumferential and axial residual stress along a radial path of the ring before and after the roll-bending process were analyzed in this study. Based on the analysis, the influence of roll-bending on the quenching residual stress in the ring was obtained.

Firstly, considering the effect of single factor on stress reduction rate, a series of roll-bending simulations of the large ring were carried out. On this basis, a set of orthogonal simulation experiments were carried out, and a set of optimum roll-bending parameters for residual stress relief was obtained.

### 4.1. Distribution of Quenching Residual Stress

**Figure 6:**
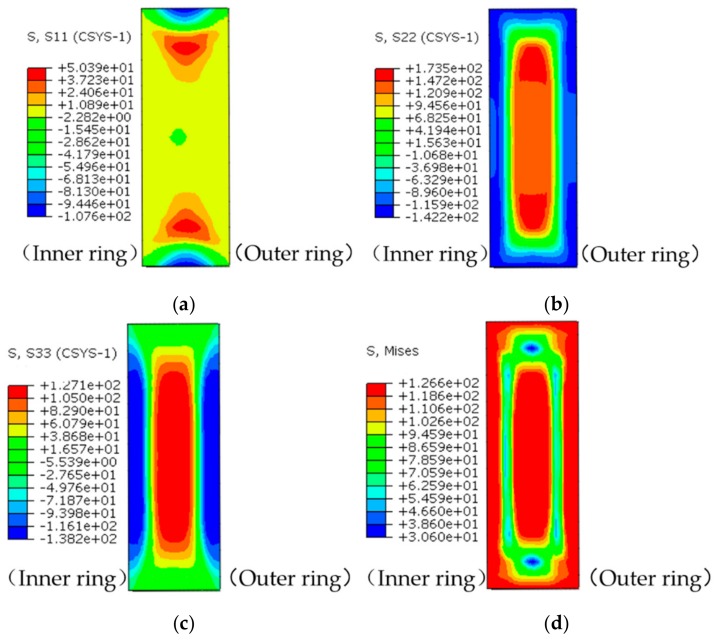
Maps of residual stress distribution after quenching. (**a**) Radial stress; (**b**) Circumferential stress; (**c**) Axial stress; and (**d**) Mises stress.

The maps of quenching residual stress on the analyzed section are shown in Figure 6. The radial stress of the ring is about −100 MPa at the upper and lower end surfaces with a diffused corrugated shape between the two surfaces, and the stress of other portions are low as shown in Figure 6a. It can be seen from the Figure 6b–d that the circumferential stress, axial stress, and Mises stress of the ring are symmetrically distributed along the geometric center of the analyzed section. Both the circumferential stress and the axial stress of the ring consist of tensile stress in the core region and compressive stress at the surface. The peak compressive and tensile stress are −140 MPa and 130 MPa, respectively. The axial stress has a relatively small value at the upper and lower edges, and the axial core tensile stress is smaller than the circumferential core tensile stress. The Mises stress distribution ranges from 40 to 140 MPa.

### 4.2. Influence of Roll-Bending on Residual Stress

Using the established FEM model, the effect of radial feed quantity (*L*), friction coefficient (*f*), and roller rotational speed (*v*) on residual stress of the ring was studied, and the reduction rates of residual stress using different roll-bending parameters were computed.

#### 4.2.1. Influence of Radial Feed Quantity on Residual Stress Reduction

Using the FEM model, the influence of radial feed quantity on the quenching residual stress of the ring was studied. In the numerical model, different radial feed quantity of 8 mm, 10 mm, 12 mm, 14 mm, and 16 mm were selected for comparative analysis, with the driven roller rotation speed of 0.3 rad/s and friction coefficient of 0.3.

**Figure 7:**
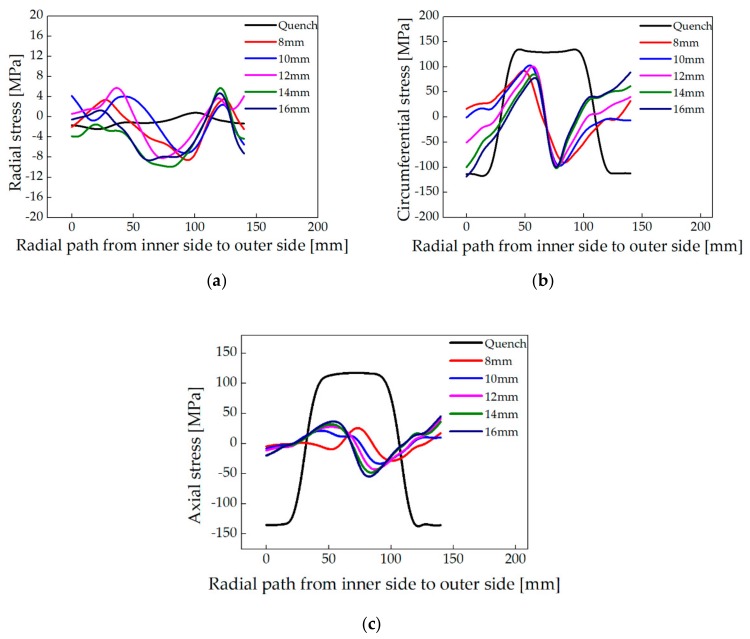
Distribution of residual stress along Line 1 after roll-bending with different radial feed quantities. (**a**) Radial stress; (**b**) Circumferential stress; and (**c**) Axial stress.

The distribution of radial, circumferential, axial, and Mises stress along Line 1 (shown in Figure 5) of the ring was obtained, as shown in Figure 7. It can be seen from the result that the radial stress before and after roll-bending is small and the change is small. The circumferential stress of different radial feed quantities is N-shaped, and the circumferential stress magnitude at the inner surface of the ring is similar to that at the outer surface, but the sign is opposite. With the increase of the radial feed quantity, the stress at the inner surface of the ring decreases gradually from tensile stress to compressive stress, while the stress at the outer surface decreases gradually from compressive stress to tensile stress. There is a little change in the circumferential stress at the center of the selected section as the radial feed quantity changes. The change of axial residual stress caused by different radial feed quantities mainly compose at the center and outer side of the selected section, and the variation range is within ±50 MPa.

Since no significant variation in the radial residual stress, the further analysis focuses on the effect of roll-bending parameters on the circumferential stress and axial stress. 

In the actual production process, the residual stress in the workpiece should be as small as possible, and the workpiece with zero residual stress is the most ideal state. In this work, the reduction of quenching residual stress of the ring can be expressed by an average reduction rate, as shown in Equation (1). Firstly, the ring was divided into *n* layers along the Line 1 direction, and the reduction rates of residual stress in each layer after roll-bending were calculated. Then, the reduction rates of residual stress in the *n* layers were averaged as average reduction rates of residual stress, i.e. Equation (1).

(1) $$\delta =\frac{\sum_{i=1}^{n}\left(\frac{\left|RS_{qi}\right|-\left|RS_{ri}\right|}{\left|RS_{qi}\right|}\right)}{n}\times 100\%$$

*δ* —Average reduction rate;
*RS_r_*—Residual stress after roll-bending;
*RS_q_*—Residual stress after quenching;
*n*—number of Layers.

After roll-bending with different radial feed quantities, the average reduction rates of circumferential and axial quenching residual stress in the large ring are shown in Table 4. It can be observed that, the highest circumferential stress reduction rate of 61.05% was obtained when the radial feed quantity is 12 mm. The average reduction rate decreases as the radial feed quantity decreases below 12 mm in descending order, and also decreases as the radial feed quantity increases above 12 mm in ascending order. The average reduction rate of the axial residual stress is above 70% with different radial feed quantities (from 8 to 16 mm), which is better than that of circumferential stress. Considering the reduction effect of circumferential and axial residual stress of the ring, the residual stress reduction effect is better when the radial feed quantity is 12 mm.

#### 4.2.2. Influence of Friction Coefficient on Residual Stress Reduction

Using the FEM model, the influence of the friction coefficient on the quenching residual stress of the ring was studied. In the finite element model, the contact friction between the feed roller and the ring, and between the driven roller and ring were set as coulomb friction, and various friction coefficients were used. The selected friction coefficients were 0.1, 0.2, 0.3, and 0.4, which were used for comparative analysis, the radial feed quantity of the feed roller was set as 12mm, and the roller speed was set as 0.3 rad/s. 

**Figure 8:**
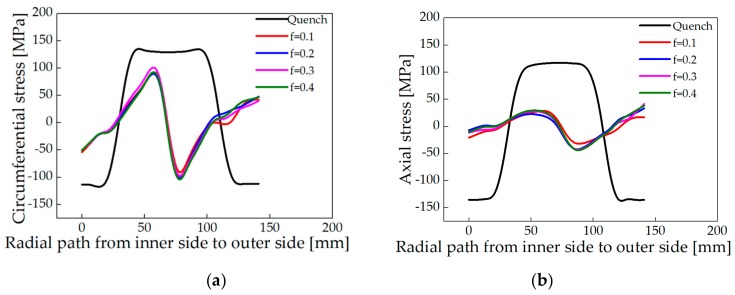
Distribution of residual stress along Line 1 after roll-bending with different friction coefficients. (**a**) Circumferential stress and (**b**) Axial stress.

After roll-bending with different friction coefficients, the distribution of circumferential and axial stress along Line 1 of the ring was shown in Figure 8. The circumferential stress of different friction coefficients is N-shaped, and the circumferential stress magnitude on the inner surface of the ring is similar to that on the outer surface, but the sign is opposite. The distribution of axial stress is similar to the circumferential stress, but the axial stress magnitude is smaller. The average reduction rates of circumferential and axial residual stress are shown in Table 5. It can be seen from the table that the average reduction rate of circumferential residual stress of the ring is above 60% when the friction coefficient changes from 0.1 to 0.4, and the difference is small. While the average reduction rate of axial residual stress is above 74% when the friction coefficient changes from 0.1 to 0.4.

In addition, it can be observed from the computed results that the circumferential stress of the ring still is high after roll-bending, while the axial stress becomes smaller. The reduction of circumferential stress deserves more attention.

#### 4.2.3. Influence of Roller Rotational Speed on Residual Stress Reduction

In the actual production process of the roll-bending process, the rotational speed of the driven roller plays an important role in reducing the residual stress of the ring. In this section, the influence of rotational speed of the driven roller on the effect of residual stress reduction was analyzed. 

In the numerical model, different rotational speeds of the driven rollers of 0.2 rad/s, 0.3 rad/s, 0.4 rad/s, and 0.5 rad/s were selected for comparison, the radial feed quantity and friction coefficient set as 12 mm and 0.1, respectively.

**Figure 9:**
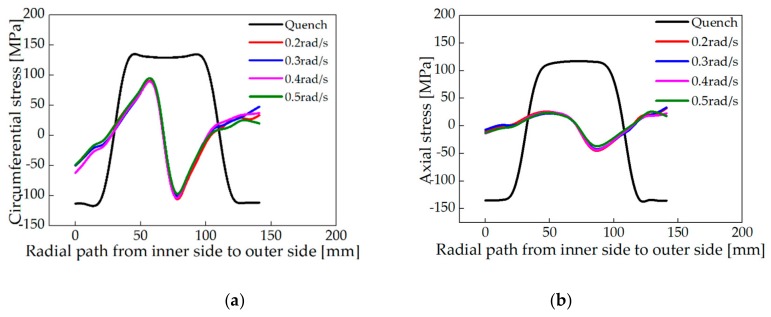
Distribution of residual stress along Line 1 after roll-bending with different roller rotational speeds. (**a**) Circumferential stress and (**b**) Axial stress.

After roll-bending with different rotation speeds (0.2, 0.3, 0.4, and 0.5 rad/s), the distribution of circumferential and axial stress along Line 1 of the ring was shown in Figure 9. The distribution of axial and circumferential stress is similar to Section 4.2.2., except for the stress magnitude. The average reduction rates of circumferential and axial residual stress are shown in Table 6. It can be seen that the average reduction rate of the circumferential residual stress of the ring is 64.94% when the rotation speed is 0.3 rad/s, while the average reduction rate of the axial residual stress of the ring is 76.70%.

### 4.3. Optimization of Roll-Bending Parameters

Based on the analyzed influences of the roll-bending parameters on residual stress, a set of orthogonal simulations were conducted. In the orthogonal simulations, different radial feed quantities of 8 mm, 10 mm, 12 mm, and 14 mm were selected, different friction coefficients of 0.1, 0.2, 0.3, and 0.4 were selected, and different rotational speeds of the driven rollers of 0.2 rad/s, 0.3 rad/s, 0.4 rad/s, and 0.5 rad/s were selected.

The analysis shows that the sensitivity of the three main parameters to the reduction rate of the ring residual stress can be ranked as follows: radial feed quantity is the first, friction coefficient is the second, and driven roller rotational speed is the third, as shown in Table 7.

The axial residual stress reduction rate is above 75%, so we mainly consider a set of roll-bending optimization parameters that satisfy the maximum reduction rate of circumferential stress. Considering that the circumferential stress of the ring is rather large, the ideal situation of residual stress controlling should be that the circumferential stress reduction rate is the largest, and the axial stress reduction rate is more than 75%. According to the results of Table 7, a set of optimized roll-bending parameters for residual stress reduction were obtained: radial feed quantity *L* = 10 mm, friction coefficient *f* = 0.1, and driven roller rotational speed *v* = 0.4 rad/s.

Then a ring roll-bending simulation was performed using the optimized parameters. The simulation result shows that the reduction rates of circumferential stress and axial stress of the ring’s inner region are 97.23% and 95.52%, respectively. The reduction rates of the circumferential and axial stress in the central layer of the selected section are 72.16% and 66.32%, respectively. Furthermore, the reduction rates of the circumferential and axial stress of the ring outer surface are 81.19% and 94.98%, respectively. While the reduction rates of the peak circumferential and axial stress are 37.56% and 73.63%, respectively. Then, average reduction rates of circumferential and axial stress of the ring are 61.72% and 86.24% respectively.

### 4.4. Comparison of Measurement Results with FEM

**Figure 10:**
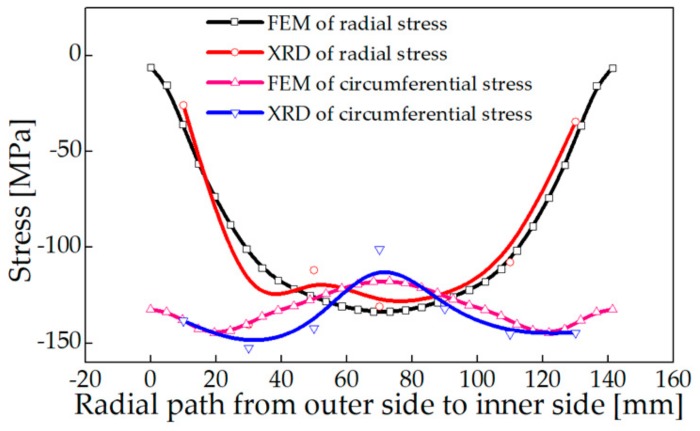
Surface stress on the axial end face of the ring.

**Figure 11:**
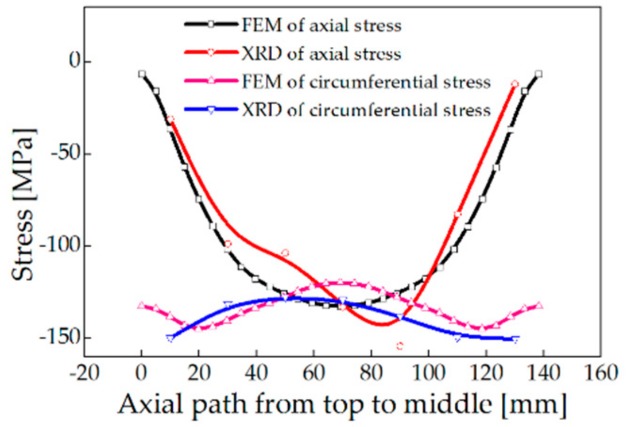
Surface stress on the inner cylindrical surface of the ring.

The test results indicated that the radial stress amplitude from the outer side to the inner side increases from −25 to −130 MPa, and then decreases to −34 MPa. The stress peak appears at the center of the thickness. The circumferential stress amplitude from the outer side to the inner side fluctuates from −101 to −152 MP, and the maximum stress occurs at 25 to 30 mm from the surface. The stress distribution at the inner cylindrical area of the large ring is similar to the stress distribution on the end surface. Measurement result and FEM result of surface stress are in good agreement as shown in Figure 10 and Figure 11, which verified the feasibility and reliability of the numerical model.

## 5. Discussion on the Evolution of Quenching Stress during the Roll-Bending Process

**Figure 12:**
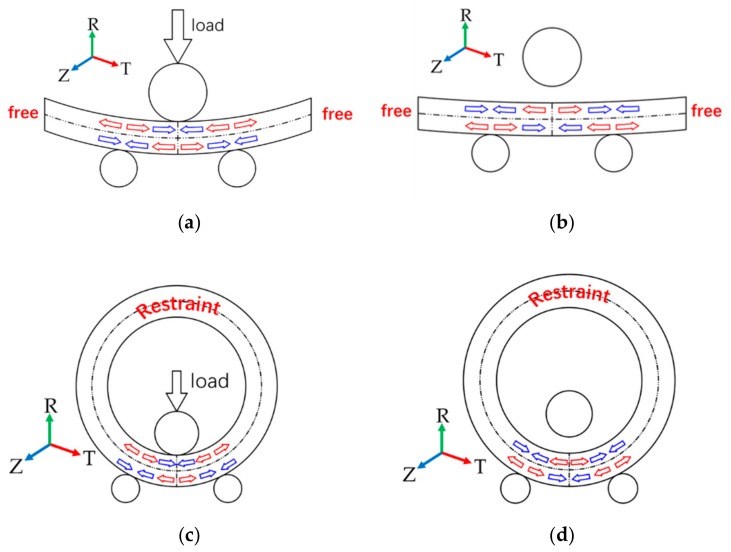
The Stress relief process of the beam and the ring (red arrow indicates tensile stress; blue arrow indicates compressive stress). (**a**) Process of feed roller pressed down for the three-roller bending beam; (**b**) Process of feed roller lifting for the three-roller bending beam; (**c**) Process of feed roller pressed down for the ring; and (**d**) Process of feed roller lifting for the ring.

Roll-bending process of ring is similar to three-roller bending process of beam, as shown in Figure 12. It could introduce tensile residual stress at the material surface and compressive residual stress in the mid-plane, as described by Abvabi [19]. While the surface of 2219 Al alloy ring presented compressive residual stress and the mid-plane presented tensile residual stress, after quenching. Therefore, roll bending was suitable for reducing the quenching residual stress of ring components.

**Figure 13:**
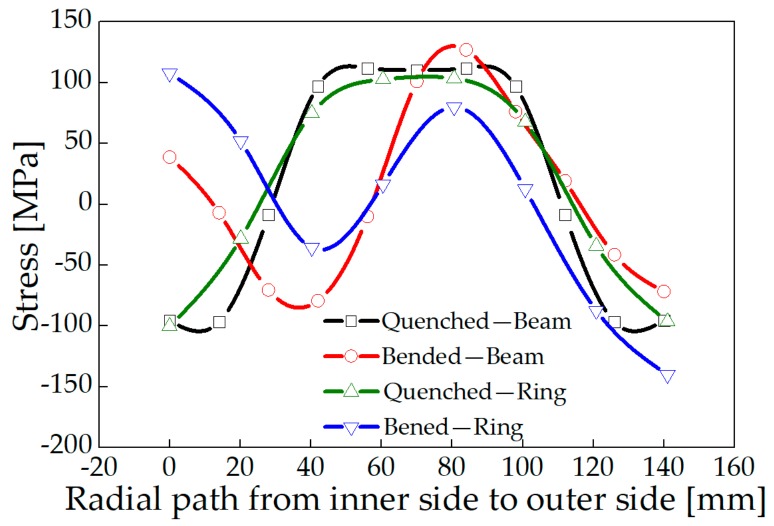
Circumferential stress of the ring and the three-roller bending beam.

**Figure 14:**
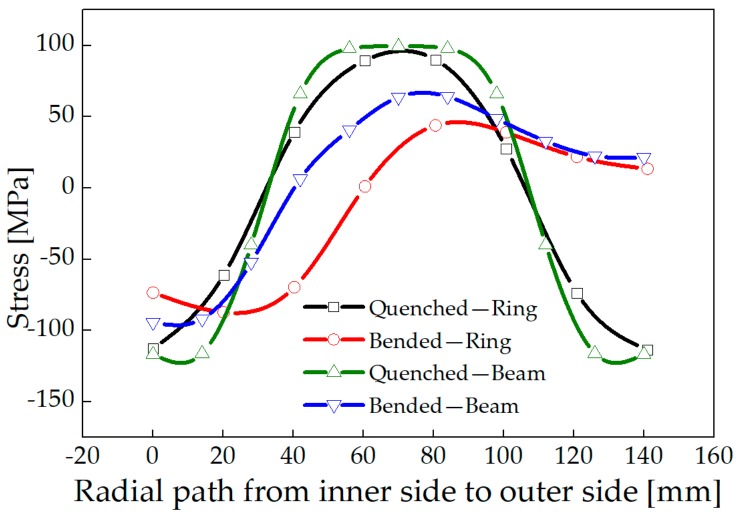
Axial stress of the ring and the three-roller bending beam.

**Figure 15:**
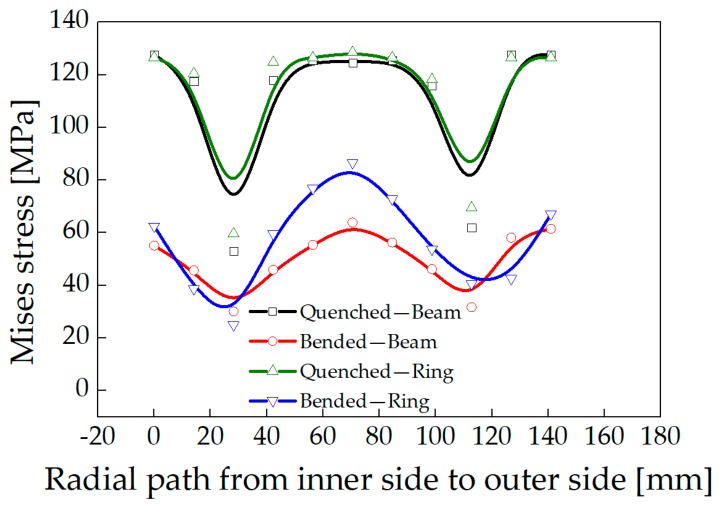
Mises stress of the ring and the three-roller bending beam.

**Figure 16:**
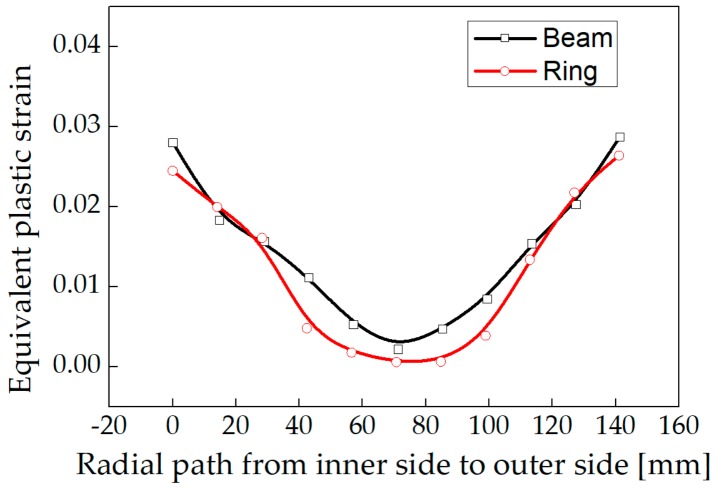
Distribution of equivalent plastic strain.

A model was established for stress relief simulation by using one-third of the ring as a simplified three-roller bending beam model. In addition, the stress distributions of a roll-bended ring and a three-roller bended beam were compared. Plastic deformation occurs in the ring after the feed roller pressed down, resulting in residual stress redistribution. The stress distribution was consistent with the study of Peng [20]. The distribution of circumferential stress and axial stress changes from arch-shaped into inverse N-shaped, which is consistent with the stress distribution trend of the three-roller bending beam, as shown in Figure 13 and Figure 14. The Mises stress distributions of the ring and the beam were shown in Figure 15, and the equivalent plastic strain distributions along the thickness path were shown in Figure 16. The W-shaped distributions of Mises stress and V-shaped distributions of equivalent plastic strain, were consistent with the research of Ktari [21]

Within 20 mm of the surface layer, the circumferential stress of the ring is 70 MPa larger than the circumferential stress of the three-roller bending beam. The axial stress of the ring is between 8 to 20 MPa smaller than the axial stress of the three-roller bending beam as shown in Figure 13 and Figure 14. The results showed that the circumferential residual stress reduction rates were 31.3% and 42.1%, and the axial residual stress reduction rates were 22.8% and 32.0%, for the ring and the beam respectively. The reason of the difference is that larger deformation occurred in the beam than the ring, as shown in Figure 16. Plastic deformation was introduced rather easily in the three-roller bending beam, while the plastic deformation in the roll-bending ring was more restricted by surrounding materials.

## 6. Conclusion

The effect of the roll-bending process for reducing residual stress of large 2219 Al alloy rings was investigated in this work. Combined with finite element simulation and experiment, the residual stress distributions of the large 2219 Al alloy ring, after quenching and roll-bending, were studied. The main points are conducted as follows:

(1) After quenching, the radial, circumferential, and axial stress of the ring were symmetrically distributed, and the radial stress is relatively small. The circumferential and axial stress were observed to be compressive at the outer surface and tensile at the core region, with the stress values between −150 and 150 MPa.
(2) The effect of radial feed quantity, friction coefficient and rotational speed on the residual stress was analyzed. In addition, a set of optimized roll-bending parameters was obtained: radial feed quantity of 10 mm, friction coefficient of 0.1, and driven roller rotational speed of 0.4 rad/s. The average residual stress reduction rates of the ring in the circumferential and axial direction reached 61.72% and 86.24% based on the optimized parameters, respectively.
(3) The roll-bending method proposed in this paper can effectively reduce the quenching residual stress of a large-size 2219 Al alloy ring. In addition, the method may be used as a reference option for residual stress relief of other ring type components.
(4) The residual stress-relief effect on the ring was compared with the effect on the three-roller bending beam. The circumferential residual stress reduction rate was 31.3% in the ring, less than 42.1% of the three-roller bending beam, and the axial residual stress reduction rate was 22.8% in the ring, less than 32.0% of the three-roller bending beam.

## Figures and Tables

**Table 1:** Compositions of 2219 Al alloy (wt. %).

Cu	Mn	Si	Zr	Fe	Mg	Zn	Ti	Al
5.8~6.8	0.2~0.4	0.2	0.1~0.25	0.3	0.02	0.1	0.02~0.1	Balance

**Table 2:** XRD measurement parameters.

X-ray Diffraction Parameters	Specification/Values
Tube type	Cr
Supplied current during the experiment	6.7 mA
Supplied voltage during the experiment	30 kV
Exposure time for the calibration	8 s
Exposure time for measurement	10 s
Collimator diameter	3 mm
Collimator distance	10.390 mm
Detector distance	50 mm
Tilt angle	−45° to 45°
Number of tilts	5/5
Rotation angle	0° to 90°
Number of rotations	2
Stress resolution	±10 MPa

**Table 3:** Thermo-mechanical properties of Al 2219 at different temperatures.

Temperature/K	293	373	473	573	673	813
Conductivity/W·(m·K)^−1^	159	169	176	180	180	180
Specific heat/J·(kg·K)^−1^	834	838	880	964	1090	1337
Young’s Modulus/MPa	71,000	65,193	56,262	37,980	31,200	25,000
Poisson’s Ratio	0.33	0.33	0.33	0.33	0.33	0.33
Yield Stress/MPa	107.79	103.42	100.98	71.67	25.36	13.10

**Table 4:** Residual stress reduction rates with different radial feed quantity.

Radial Feed Quantity/mm	8	10	12	14	16
reduction rate of circumferential stress	48.69%	54.00%	61.05%	47.15%	41.11%
reduction rate of axial stress	82.24%	78.96%	75.36%	76.25%	74.29%

**Table 5:** Residual stress reduction rates with different friction coefficient.

Friction Coefficient	0.1	0.2	0.3	0.4
reduction rate of circumferential stress	64.94%	61.31%	61.05%	61.42%
reduction rate of axial stress	76.70%	77.40%	75.36%	74.69%

**Table 6:** Residual stress reduction rates with different roller rotational speeds.

Roller Rotational Speed (rad/s)	0.2	0.3	0.4	0.5
reduction rate of circumferential stress	60.15%	64.94%	62.46%	61.26%
reduction rate of axial stress	80.75%	76.70%	76.02%	78.58%

**Table 7:** Orthogonal simulation of the ring roll-bending (three factors and four levels).

Experimental Numbers	*L*(mm) (A)	*f* (B)	*v* (rad/s) (C)	RCS	RAS
1	1(8)	1(0.4)	1(0.2)	29.22%	84.17%
2	1	2(0.1)	2(0.3)	51.10%	85.00%
3	1	3(0.2)	3(0.4)	40.62%	83.83%
4	1	4(0.3)	4(0.5)	30.27%	87.99%
5	2(10)	1	2	58.24%	88.80%
6	2	2	3	61.72%	86.24%
7	2	3	4	56.06%	87.25%
8	2	4	1	53.74%	87.37%
9	3(12)	1	3	51.90%	88.57%
10	3	2	4	55.09%	85.32%
11	3	3	1	51.94%	86.85%
12	3	4	2	54.18%	85.68%
13	4(14)	1	4	32.42%	78.50%
14	4	2	1	28.30%	79.99%
15	4	3	2	20.63%	75.75%
16	4	4	3	30.67%	82.02%
k1	1.512	1.718	1.632		
k2	2.298	1.962	1.841		
k3	2.131	1.692	1.849		
k4	1.120	1.689	1.738		
Range	0.295	0.068	0.054		
COP	A2	B2	C3		
k1	3.410	3.400	3.384		
k2	3.497	3.365	3.352		
k3	3.464	3.337	3.407		
k4	3.163	3.431	3.391		
Range	0.084	0.024	0.014		
AOP	A2	B4	C3		

Comment: RCS is reduction rate of circumferential stress; RAS is reduction rate of axial stress; COP is a set of circumferential optimization parameters; AOP is a set of axial optimization parameters.
